# Extracellular vesicle‐mediated regulation of tumor angiogenesis— implications for anti‐angiogenesis therapy

**DOI:** 10.1111/jcmm.16359

**Published:** 2021-02-14

**Authors:** Shuqiong Zhang, Junyao Yang, Lisong Shen

**Affiliations:** ^1^ Department of Clinical Laboratory Xinhua Hospital Shanghai Jiao Tong University School of Medicine Shanghai China

**Keywords:** angiogenesis, drug resistance, endothelial cells, extracellular vesicles, non‐coding RNA

## Abstract

Angiogenesis plays an important role in tumour progression. However, anti‐angiogenesis therapy of inhibiting pro‐angiogenic factors failed to meet expectations in certain types of tumour in clinical trials. Recent studies reveal that tumour‐derived extracellular vesicles (EVs) are essential in tumour angiogenesis and anti‐angiogenesis drug resistance. This function has most commonly been attributed to EV contents including proteins and non‐coding RNAs. Here, we summarize the recent findings of tumour‐derived EV contents associated with regulating angiogenesis and illustrate the underlying mechanisms. In addition, the roles of EVs in tumour microenvironmental cells are also illustrated with a focus on how EVs participate in cell‐cell communication, contributing to tumour‐mediated angiogenesis. It will help offer new perspectives on developing targets of anti‐angiogenesis drugs and improve the efficacy of anti‐angiogenesis therapies based on tumour‐derived EVs.

## 1 INTRODUCTION

Blood vessels are essential to every part of the body including tumours by delivering oxygen and nutrients.^1^ Normally, endothelial cells (ECs), which line the inner surface of blood vessels, are involved in tissue growth and repair.^2^ However, tumour vessels are like ‘mosaic vessels’, which are composed of ECs and tumour cells, displaying abnormal structure and function with the seemingly chaotic organization.^3^ There is a significant correlation between the degree of angiogenesis and malignancy.^4^ Therefore, the studies about the regulation mechanism of tumour angiogenesis are of great importance to the development of therapeutic strategies against tumour.^5^

Tumour angiogenesis is related to the division, proliferation and migration of vascular ECs. In the process of capillary sprouting, tip cells, which are highly migratory but non‐proliferative ECs, guide the new sprout growing in the right direction. Stalk cells, with high proliferative capacity, elongate the new sprout.^6^ Several pro‐angiogenic factors have been found to initiate neovascularization.

Up to date, for the vital importance of VEGF, researchers have focused on developing VEGF and VEGFR inhibitors as anti‐angiogenic drugs.^7^ However, clinical trials to anti‐angiogenesis therapy by inhibiting pro‐angiogenic factors failed to meet expectations. Therefore, studies are required to illuminate the resistance mechanism and develop additional anti‐angiogenic drugs according to angiogenesis targets besides VEGF signalling. So far, among the alternative therapeutic strategies and studies, extracellular vesicles (EVs) are reported to have a crucial role in tumour angiogenesis and anti‐angiogenesis drug resistance.

According to the EV biogenesis mechanisms, EVs can be classified into two broad categories, referred to here as exosomes and shed microvesicles.^8^ EVs can derive from the endosomal compartment (exosomes) or shed from the plasma membrane (microvesicles, oncosomes and apoptotic bodies).^9^ EVs mediate intercellular communication between diverse cells and thereby resulting in the impact on normal and pathological conditions.^10^ It is due to the abundant molecules that EVs carry such as proteins and peptides, lipids, RNA and DNA, making recipient cells profound phenotypic variations in the tumour microenvironment.^8^, ^11^ EVs have increasingly become the focus of potential targets for novel therapeutic strategies. Moreover, some tumour cell–derived EVs are reported to promote tumour angiogenesis by inducing cancer‐associated fibroblast (CAF) differentiation in stromal cells or normal fibroblasts via a complex mixture of small RNAs and proteins. Besides, recent studies revealed that cancer stem cells (CSCs) play a key role in tumour angiogenesis. More importantly, it is observed that treatments with anti‐angiogenesis drugs interact with EVs and induce alteration of tumour‐derived EVs, which are related to tumour progression and drug resistance. Therefore, anti‐angiogenic strategies targeting on tumour cell–derived EVs could represent new approaches for the treatment of tumour development.^11^, ^12^

Here, we review the currently available data to clarify the mechanisms of intercellular communication these EVs participate in and the consequences of molecular transfer in EVs for tumour angiogenesis and anti‐angiogenesis drug resistance. It may help provide the researchers with new perspectives on developing alternative anti‐angiogenesis drug and improve the efficacy of anti‐angiogenesis therapies.

## 2 TUMOUR‐DERIVED EVS REGULATING ANGIOGENESIS VIA NON‐CODING RNAS AND PROTEINS

Accumulating studies reveal that tumour‐derived EVs can transport components including non‐coding RNAs such as circular RNAs (circRNAs), long non‐coding RNAs (lncRNAs), microRNAs (miRNAs) and proteins, thereby offering novel biomarkers and therapeutic targets on tumour angiogenesis.

### 2.1 The angiogenic related non‐coding RNAs in tumour‐derived EVs

Recently, further studies have highlighted the role of non‐coding RNAs from tumour‐derived EVs in the regulation of tumour angiogenesis.^5^, ^13^

#### 2.1.1 circRNAs

circRNAs, formed via back splicing, are single‐stranded RNAs with covalent bonding. In eukaryotes, most of the circRNAs are non‐coding RNAs acting as microRNA or protein inhibitors, but some encode peptides with tissue‐specific and cell‐type–specific expression patterns.^14^ circRNAs are mostly found in the cytoplasm with a long half‐life.^15^ They do not have the typical 5 'cap and 3' poly(A) tail‐like mRNAs and cannot be degraded easily.^16^ Also, it has been shown that circRNAs are enriched and stable in EVs, regulating cellular biological processes, and probably to be biomarkers for different pathological conditions.

**FIGURE 1:**
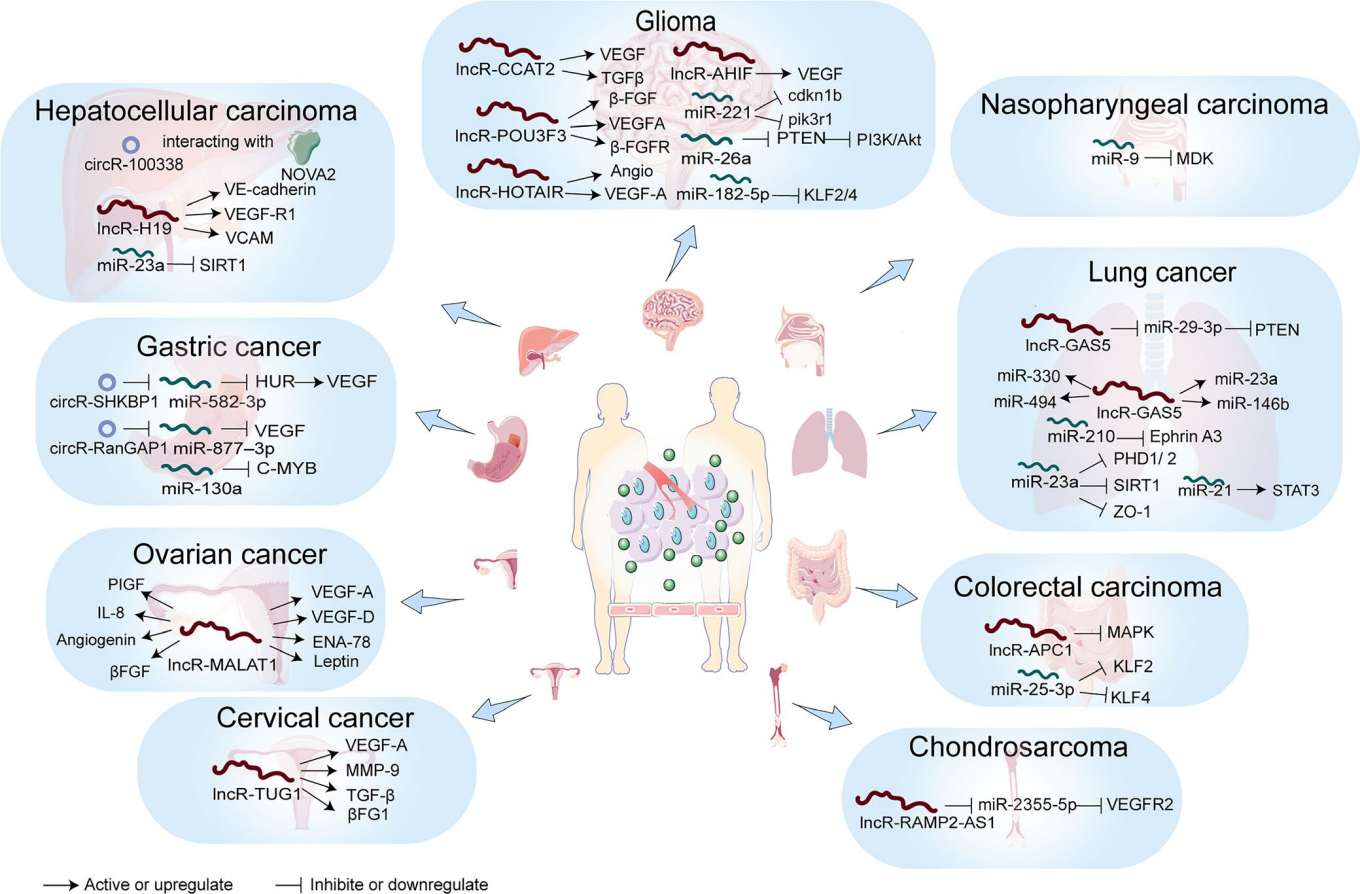
Functional mechanisms of the non‐coding RNAs in tumour‐derived EVs regulating angiogenesis in different types of tumours

Recently, some circRNAs from tumour‐derived EVs are found to be related to poor prognosis and advanced tumour node metastasis classification stage, making them potential RNA biomarkers. For example, circ‐SHKBP1 and circ‐RanGAP1 in gastric cancer (GC) are reported to be up‐regulated and effectively delivered from GC‐derived EVs into the circulation. circ‐SHKBP1 from GC EVs promotes cancer progression by regulating the miR‐582‐3p/HUR/VEGF axis and suppressing the degradation of HSP90.^17^ GC tissues and peripheral blood of patients are found to be rich in circ‐RanGAP1. Mechanistically, circ‐RanGAP1, acting as a ceRNA, inhibits miR‐877‐3p, thus making an increased expression of *VEGFA*.^18^ EV circRNA‐100,338 is persistently highly expressed in serum of hepatocellular carcinoma (HCC) patients who had the operation of curative hepatectomy may predict lung metastasis and poor survival.^19^ What's more, internalized EV circRNA‐100,338 in ECs might regulate the angiogenesis by interacting with an RNA‐binding protein, NOVA2. These findings reveal a novel mechanism and an alternative therapeutic strategy of circRNAs for tumours (Figure 1).

#### 2.1.2 lncRNAs

lncRNAs, greater than 200 nucleotides in length, exert essential biological functions by interacting with many molecules in both cytoplasm and nuclei.^20^ It is extensively reported that lncRNAs are involved in transcriptional regulation. EV lncRNAs that can be found in different types of body fluids are highly stable, thus making them promising biomarkers for tumour liquid biopsy.

Emerging evidence suggests that EV lncRNAs promote the key pro‐angiogenesis gene and protein expression, cell proliferation and drug resistance by acting as ceRNA or binding mRNA.^21^ For example, CD90^+^ liver cancer cells’ EVs containing lncRNA‐H19 induce an obvious increase in the *ICAM1* and *VEGF* expression, leading to increasing ability of ECs to arrange in vitro tubular‐like structures and promote heterotypic adhesion between ECs and CSC‐like liver cells.^22^ The lncRNA‐APC1 expression is decreased in colorectal carcinoma EVs. lncRNA‐APC1 directly binds *Rab5b* mRNA, thereby reducing its stability and ultimately leading to decreased EV production. This action also inhibits the overactivation of the MAPK pathway in ECs and the subsequent suppression of angiogenesis.^23^ Epithelial ovarian cancer cells can accelerate angiogenesis through the regulation of EV‐mediated transfer of lncRNA‐MALAT1 to ECs, regulating well‐known pro‐angiogenic genes.^24^ Cervical cancer cells transferred lncRNA‐TUG1 to ECs.^25^ The lncRNA‐p21 expression defines prognosis in non–small cell lung cancer and modulates endothelial cell behaviour EVs..^26^ The lncRNA‐GAS5 in the angiogenesis is supposed to be fulfilled by competitively binding miR‐29‐3p with PTEN.^27^ In chondrosarcoma, mechanistically, EV lncRNA‐RAMP2‐AS1 regulates *VEGFR2* expression by acting as a ceRNA of miR‐2355‐5p.^28^ In glioma, lncRNA‐AHIF is the natural antisense transcript of hypoxia‐inducible factor‐1α (HIF‐1α) and is exactly complementary to the 3'‐untranslated region of HIF‐1α mRNA.^29^ Increased lncRNA‐AHIF expression has been observed to be in parallel with that of VEGF.^30^ lncRNA‐HOTAIR enhances angiogenesis by induction of *VEGFA* expression.^31^ lncRNA‐CCAT2 activates VEGF and TGF‐β in ECs.^32^ lncRNA‐POU3F3 increases the expression of FGF2, VEGFA, bFGFR and Angio^33^ (Figure 1).

#### 2.1.3 miRNAs

miRNAs, containing about 23 nucleotides, play an important role in gene regulation by directing protein‐coding genes post‐transcriptional repression via pairing to the mRNAs.^34^ So far, it is revealed that miRNAs are important in diverse biological processes of cancer, including tumour growth and drug resistance.^35^ Extensive studies have revealed that miRNAs are involved in tumour angiogenesis by regulating the activity of ECs.^13^ Importantly, with the development of RNA delivery technology, miRNA‐based interventions may become novel therapy to target tumour angiogenesis.^5^

Several miRNAs from tumour‐derived EVs have been reported to act as predictive non‐invasive biomarkers for anti‐angiogenic therapy.^5^, ^36^ For example, miRNA‐23a from HCC‐derived EVs induces the process of angiogenesis by targeting SIRT1 in the recipient ECs.^37^ miR‐130a in gastric cancer promotes angiogenesis via targeting C‐MYB.^38^ miR‐25‐3p from colorectal cancer cell–derived EVs regulates the expression of VEGFR2, ZO‐1, occludin and Claudin 5 in ECs by targeting KLF2 and KLF4.^39^ In glioma, miR‐26a promotes angiogenesis by targeting PTEN and thereby activates the PI3K/Akt signalling pathway.^40^ miR‐221 promotes tip cell behaviour through repression of two targets: *cdkn1b* and *pik3r1*.^41^ EV miR‐182‐5p directly suppressed its targets KLF2 and KLF4, leading to the accumulation of VEGFR.^42^ In lung cancer, miR‐21 from EVs leads to STAT3 activation, which increases VEGF levels in recipient cells.^43^ Besides, miR‐210 can lead to a down‐regulation of ephrin A3, which is miR‐210 target protein.^44^ miR‐23a activates angiogenesis and vascular permeability by targeting prolyl hydroxylase and tight junction protein ZO‐1.^45^ miR‐9, which inhibits angiogenesis by targeting MDK and regulating the PDK/AKT pathway, is found to be reduced in EVs derived from cultured NPC cells and plasma samples^46^ (Figure 1).

### 2.2 The angiogenic‐related proteins in tumour‐derived EVs

Up to date, it has been reported that multiple molecules and mechanisms (eg VEGF/ VEGFR2 signalling,^47^ MAPK,^48^ Notch signalling^49^ and Wnt/β‐catenin signalling^50^) are involved in tumour angiogenesis. Also, accumulating studies find that tumour‐derived EVs contain proteins related to the signalling pathways mentioned above, thus making the EVs a vital role in tumour angiogenesis.

In recent years, the researchers not only identify the proteins from different types of tumour‐derived EVs, but also further study how these proteins are produced, how they exist in the EVs and how they are involved in the angiogenesis. For example, in a recent study, tumour‐derived EVs from breast cancer promote VEGF receptors and tumour angiogenesis by VEGF_90K_.^51^ VEGF_90K_ is generated by the cross‐linking of VEGF_165_, catalysed by the enzyme tissue transglutaminase and associated with MVs by the interaction with the Hsp90.^51^ And a 3D rotation of EVs displays that Hsp90 and VEGF are located on the surface of the EVs.^51^ Furthermore, it is reported recently that the angiogenic effects of tumour EVs are mainly mediated by aspartate β‐hydroxylase (ASPH) signalling.^52^ ASPH is found to guide tumour cells to secrete EVs carrying pro‐invasive/pro‐metastatic components such as active Notch receptor and ligand, and regulators ADAMs and downstream MMPs.^52^ More proteins from different types of tumour‐derived EVs and their angiogenesis mechanism are summarized in Table 1.

**TABLE 1:** Proteins from tumour‐derived EVs regulating angiogenesis

Tumour type	Cargos	Mechanism	Refs
Head and neck squamous cell carcinoma	CEMIP	Up‐regulating the cytokines encoded by Ptgs2, Tnf and Ccl/Cxcl	^54^
Hepatocellular carcinoma	EPHB2	Inducing ephrin‐B reverse signalling through STAT3	^55^
	Ephrin‐B2, Dll4	Mediating co‐dependence of HCC and EPC intercellular crosstalk	^56^
Glioma	LOXL4	Activating the FAK/Src pathway	^57^
	Sema3A	Recruiting myeloid cells in a Sema3A/NRP‐1–dependent manner and then secreting pro‐angiogenic factors	^58^
Breast cancer	Proteoglycans glypican‐1, syndecan‐4	Serve as co‐receptors for angiogenic factors	^53^
	VEGF_90K_	Activating VEGF receptors and tumour angiogenesis	^51^
Ovarian cancer	Annexin II	Causing macrophage mediates activation of the p38mapk, NF‐κB and STAT3 pathways and increases secretion of IL‐6 and TNF‐α	^59^
	Active Notch receptor, ligand, ADAMs and MMPs	Activating Notch signalling pathways	^52^
	Soluble E‐cadherin	Heterodimerizing with VE‐cadherin on ECs and transduce a novel sequential activation of β‐catenin and NF‐κB signalling.	^60^
Nasopharyngeal carcinoma	ATF2, MTA1, SARS, ROCK1/2	EBNA1 activating AP‐1 transcription factor pathway by binding to ATF2 and elevated expression of AP‐1 targeting VEGF; SARS and ROCK1/2 are unique regulators in angiogenesis and angiosarcoma tumour progression.	^61^
	ICAM‐1, CD44v5	Regulating several signalling pathways including ERK1/2 kinase, eNOS, MAPK, p38, RhoA/ROCK and Src kinase	^62^
Bladder cancer	HAX‐1	Binding with many cellular and viral proteins	^63^
	EDIL‐3	Activating epidermal growth factor receptor signalling	^64^

Abbreviations: ADAM, a disintegrin and metalloprotease domain; ATF2, activating transcription factor 2; CD44v5, CD44 variant isoform 5; CEMIP, cell migration–inducing and hyaluronan‐binding protein; EDIL‐3, EGF‐like repeats and discoidin domains 3; eNOS, endothelial nitric oxide synthase; EPCs, endothelial progenitor cells; EPHB2, ephrin type b receptor 2; HAX‐1, HCLS1‐associated protein X‐1; HCC, hepatocellular carcinoma; ICAM‐1, intercellular adhesion molecule‐1; LOXL4, lysyl oxidase‐like 4; MMP, matrix metalloproteinase; MTA1, metastasis‐associated 1; Ptgs2, prostaglandin‐endoperoxide synthase 2; ROCK1/2, Rho‐associated, coiled‐coil containing protein kinase 1/2; SARS, seryl‐tRNA synthetase; VEGF_90K_, 90 kD form of vascular endothelial growth factor.

## 3 TUMOUR‐DERIVED EVs REGULATING ANGIOGENESIS VIA CAF DIFFERENTIATION

CAFs can release many regulatory molecules and remodel the extracellular matrix, regulating the biological processes of between other stromal cells and tumour cells via intercellular contact.^65^ CAFs are also known to enhance angiogenesis via secretion of factors such as VEGFA, PDGFC, FGF2, osteopontin and frizzled‐related protein 2 (SFRP2).^66^ CAFs are revealed to be generated in three major ways: the transformation of residual fibroblasts; endothelial‐to‐mesenchymal transition (EndMT); and differentiation from mesenchymal stem cells (MSCs).^67^, ^68^ CAFs can derive from multiple resident precursors, such as ECs, smooth muscle cells, myoepithelial cells and MSCs.^69^ Recently, EVs from tumour cells are reported to act on residual fibroblasts or other cells to induce CAF differentiation, then promoting angiogenesis by secreting angiogenic factors, including VEGF, TGF‐β, FGF2, MMP9 and MMP2.^70^

Tumour cell–derived EVs have been reported to induce CAF differentiation from stromal cells or normal fibroblasts via a complex mixture of small RNAs and proteins. For example, intercellular contact between MSCs and cancer cells results in MSCs expressing the CAF‐like phenotype character such as FSP, α‐SMA and CXCL12.^71^, ^72^ Treating with chronic lymphocytic leukaemia (CLL) cells, EVs, the expression of several genes already associated with the CAF gene signature (eg *ICAM1*, *MMP1*), are observed in MSCs and ECs.^71^ Besides, it is proposed that increased secretion of NF‐κB signalling, cytokines and chemokines from CLL EVs reprogrammed MSCs to the CAF differentiation.^71^, ^73^ TGF‐β1–dependent fibroblast differentiation can be induced by prostate cancer‐derived EVs, which resemble stromal cells isolated from prostate cancer tissue.^74^ Furthermore, colorectal cancer cell–derived EVs facilitate to functional heterogeneity of activated fibroblasts.^75^ What's more, a recent study noted that HCC‐derived EV miRNA‐21 leads to converting hepatocyte stellate cells to cancer‐associated fibroblasts by down‐regulated PTEN and activated PDK1/AKT signalling pathway.^70^ Also, clinical data indicated that higher vessel density and greater activation of CAFs are associated with a high level of EV miRNA‐21 from HCC patients’ serum.^70^

Moreover, further studies have suggested tumour‐derived EVs transdifferentiate CAFs by EndMT, initiating angiogenic and metastatic evolution. However, although cancer‐derived EVs promoted EndMT, MSC‐derived EVs were reported to suppress EndMT, maintain vascular homeostasis and induce angiogenesis, finally resulting in CAFs back to ECs.^67^

**FIGURE 2:**
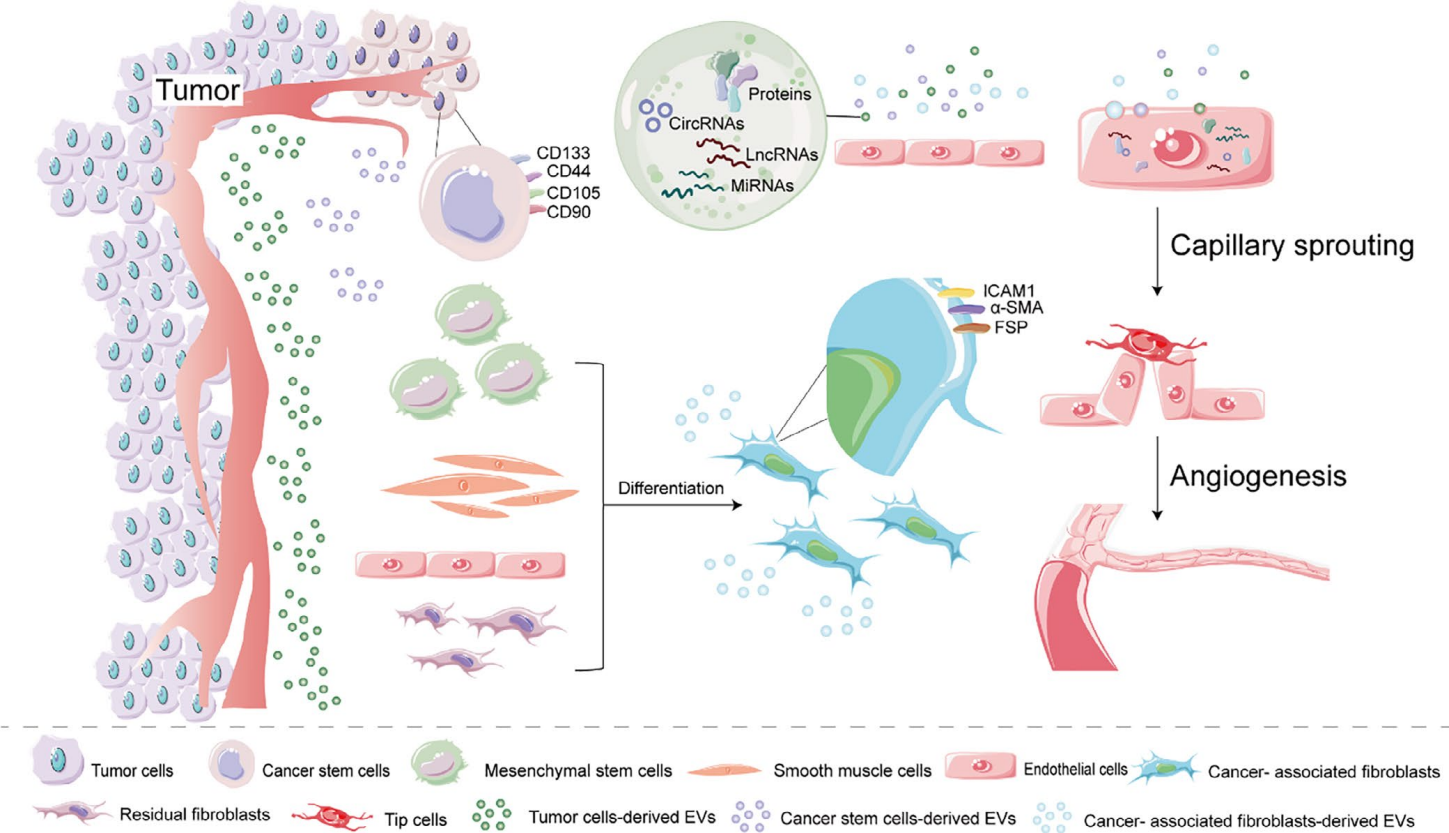
Tumour cell–derived EVs promote angiogenesis via being ingested by endothelial cells and inducing CAF differentiation. Tumour cell–derived EVs contain proteins, miRNAs, lncRNAs and circRNAs, which can be absorbed by ECs, promoting angiogenesis. CSCs, with the markers of CD133, CD90, CD44 or CD105, are also found to play an essential part in tumour angiogenic. Besides, tumour cell–derived EVs are reported to induce CAF differentiation in stromal cells, smooth muscle cells, ECs or normal fibroblasts via a complex mixture of small RNAs and proteins

Additionally, it is revealed that EVs derived from different tumour stages can activate fibroblasts displaying various expressions. For example, pro‐angiogenic and pro‐proliferative proteins are found to be increased in fibroblasts activated by early‐stage tumour‐derived EVs. In contrast, activated by late‐stage tumour‐derived EVs, fibroblasts have a greater ability to invade through the extracellular matrix^75^ (Figure 2).

## 4 CANCER STEM CELL–DERIVED EVs ACTING AS A KEY ROLE IN ANGIOGENESIS

Cancer stem cells (CSCs), with the ability to self‐replicate, differentiation and exist for a long time, are thought to have the potential to develop tumours and in particular, as the tumour metastasis, become the source of new tumour.^76^ It is still not well known how CSCs come, but some studies suggest that they are derived from either oncogenic mutations in normal stem cells or the dedifferentiation of cells to a CSC phenotype.^77^ Specially, CSC‐derived EVs also express CSC‐specific markers such as CD90, CD105, CD133 and CD44.^22^, ^78^, ^79^ Emerging evidence revealed that CSCs play a crucial part in tumour progression, including tumour angiogenic. For instance, in glioma, a highly angiogenic malignancy, the stem cells with CD44 marker promote phenotypes of cancer stem cell and radiation resistance.^80^ The CD133^+^ stem cells are related to VEGF production and angiogenic phenotype in ECs.^81^, ^82^ Also, it is reported that CSCs might participate in angiogenesis by secreting EVs with pro‐angiogenic potential. For example, CSC‐like CD90^+^ cells could promote angiogenesis by EVs containing lncRNA‐H19, thus influencing the tumour microenvironment.^22^ Glioma stem cell–derived EVs containing miR‐26a induce angiogenesis via activating the PI3K/Akt signalling pathway by targeting PTEN.^40^ In renal cancer, CD105^+^MVs from cancer stem cells contained mRNAs for growth factors such as MMP9, ephrin A3, FGF, MMP2, angiopoietin 1 and VEGF.^78^ It is also reported that mRNAs of genes related to angiogenesis are found only in CD105^+^EVs. Therefore, it suggests the cancer stem cell–derived EVs, rather than the whole tumour cell–derived EVs, promote tumour growth and invasion^78^ (Figure 2).

## 5 TUMOUR‐DERIVED EVs‐RELATED DRUG RESISTANCE

As tumour growth depends on angiogenesis, tumour blood vessels have been recognized as an important target for tumour therapy, and many anti‐angiogenesis drugs have been found and tested with good results. Up to date, the US Food and Drug Administration approves the clinical use of the anti‐angiogenic drugs targeting VEGF and several VEGF (receptor)‐based inhibitors in many types of cancer.^2^ Bevacizumab, aflibercept and ramucirumab targeting the VEGF/VEGFR signalling pathway have been used for certain tumour type therapy. Multi‐targeted tyrosine kinase inhibitors, such as sorafenib and sunitinib, also inhibit angiogenic signals. In recent studies, anti‐angiogenesis drugs have been found to alter tumour EV secretion and contents, which result in the suppression of angiogenesis, further supporting its positive effect as an anti‐tumour therapy. However, accumulating evidence indicates that monotherapy with VEGF receptor kinase inhibitors fails to take effect and even induce side effects when combined with chemotherapy. Although drug resistance has been intensely researched, the molecular mechanism of tumour resistance remains uncertain. Tumour microenvironment including EVs is revealed to play an essential part in drug resistance. Emerging evidence shows that the mechanisms to induce drug‐resistant phenotypes through EVs are the protein transfer and transmission of nucleic acids.^21^ Therefore, blocking tumour angiogenesis signals by controlling of EV secretion or alteration of EV contents may act as a novel strategy to promote the long‐term efficacy of anti‐angiogenic therapies.^83^

### 5.1 The alterations of tumour‐derived EVs under anti‐angiogenesis drugs

**FIGURE 3:**
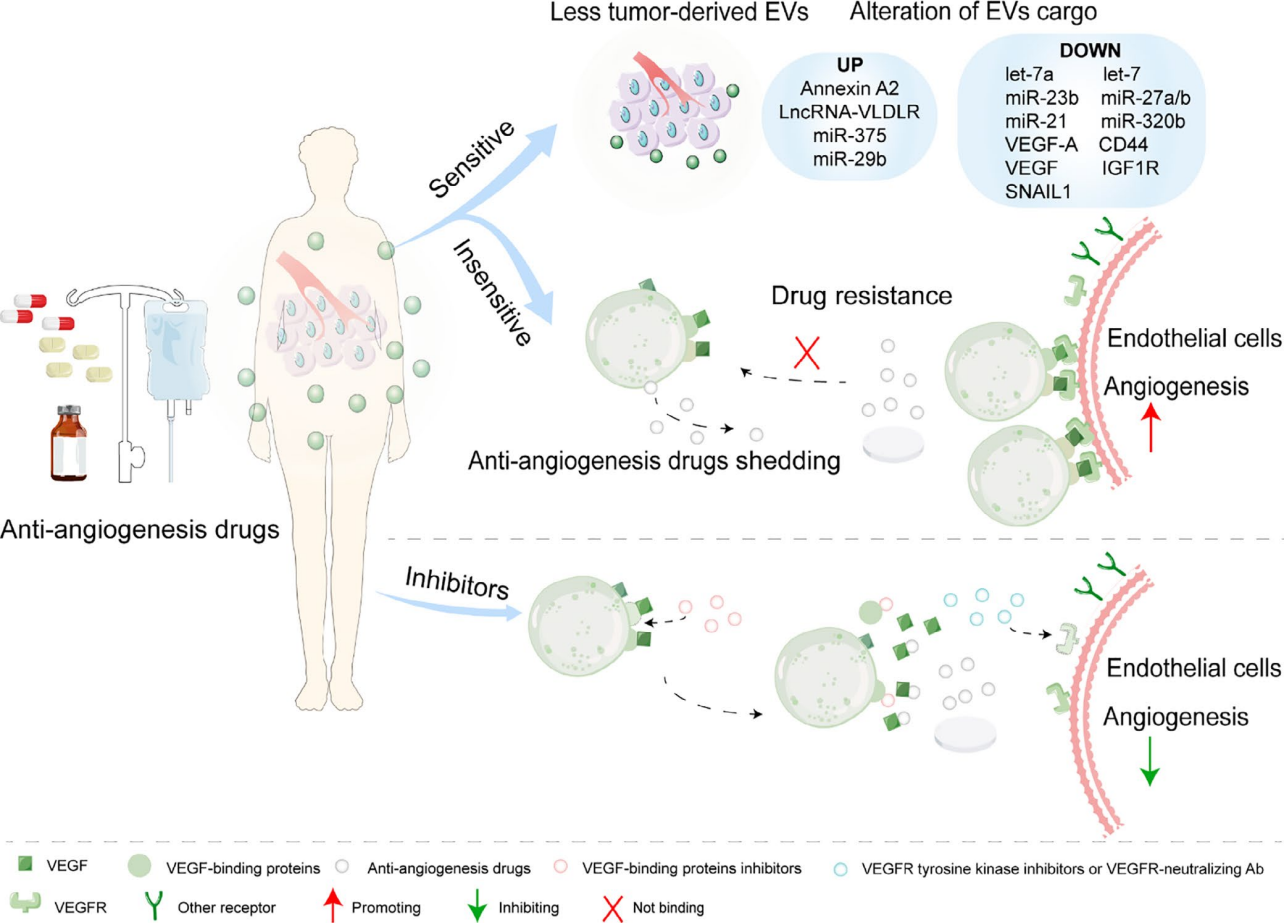
The alterations of tumour EVs under anti‐angiogenesis drugs and drug resistance. Anti‐angiogenesis drugs can reduce tumour‐derived EV secretion and alter EV contents. Drugs shedding at the surface of tumour‐derived EVs appear as a possible way for cancer cells to shield themselves against treatment. Besides, EV‐VEGF is not neutralized by bevacizumab. Treatment with a VEGF‐binding protein inhibitors releases VEGF from EVs, promoting the sensitivity of EV‐associated VEGF to anti‐angiogenesis drugs. The activity of EV‐VEGF can be blocked by VEGFR tyrosine kinase inhibitors or VEGFR2‐neutralizing antibodies. These inhibitors rather than bevacizumab might be more effective as anti‐tumour therapy for patients who have elevated EV‐VEGF levels

The contents of tumour‐derived EVs can be blocked to suppress tumour angiogenesis and offer new insight into the anti‐tumour activity of anti‐angiogenesis drugs, thereby supporting its positive use in tumour therapy.^84^ For example, a natural compound, docosahexaenoic acid with anti‐angiogenesis action, alters breast cancer EV secretion and miRNA contents, which results in the suppression of angiogenesis. Among the miRNAs that were altered in EVs, miR‐23b and miR‐320b in ECs decreased the expression of pro‐angiogenic factors and greatly suppressed tube formation.^84^ Additionally, dasatinib, a Src family kinase inhibitor, blocked EC tubular differentiation in response to tumour‐derived EVs.^85^ Moreover, both imatinib and dasatinib effectively reduced the release of chronic myeloid leukaemia cell–derived EVs, which induce angiogenesis in Src‐dependent signalling, by more than 55%.^85^ Bevacizumab, which neutralizes VEGFA secreted by tumour cells and leads to an increase in Annexin A2 with a decrease in CD44, might suggest alterations in the EVs under bevacizumab treatment.^86^ With the therapy of C6‐ceramide (a ceramide pathway activator), the level of miR‐29b was increased.^87^ C6 ceramide therapy suppresses the angiogenic activity of multiple myeloma EVs via the miR‐29b/Akt pathway. Amla extract down‐regulates pro‐angiogenic molecules via up‐regulation of cellular and EV miR‐375 in human ovarian cancer cells^88^ (Figure 3).

### 5.2 Tumour‐derived EV‐related anti‐angiogenesis drug resistance

It is also observed that treatment with anti‐angiogenesis drugs can interact with EVs, which are associated with therapeutic resistance. The emerging studies in EVs are conducive to further understanding the mechanism of anti‐angiogenesis drug resistance and improve treatment efficacy in cancer therapy.

Accumulating studies reveal that EVs play an important role in anti‐angiogenesis drug resistance. For instance, GBM cells can capture bevacizumab directly and eventually classify bevacizumab at the surface of the GBM cell–derived EVs.^86^ Normally, bevacizumab is located on the surface of EVs and is not internalized in them. However, it is reported that bevacizumab from tumour‐derived EVs could not bind to VEGFA, so there is a possibility that tumour cells escape themselves from treatment by bevacizumab shedding at the surface of tumour‐derived EVs.^86^ In addition, during incubation with sorafenib (a multikinase inhibitor, has anti‐angiogenic effects by inhibiting pro‐angiogenesis factor pathways^89^), lncRNA‐VLDLR is significantly increased in HCC cells and EVs.^89^ It revealed a novel mechanism about intercellular contact between EVs and recipient cells and regulation of target gene expression leading to acquired drug resistance. Moreover, a recent study suggests that HCC cell–derived EVs play an important role in sorafenib resistance in liver cancer cells by suppressing sorafenib‐induced apoptosis and inducing the HGF/c‐Met/Akt signalling pathway. Although the angiogenesis inhibitor, vandetanib, completely inhibits miR‐9–induced angiogenesis and promoted autophagy of ECs, it is found to result in the release of VEGF‐enriched EVs.^83^

Besides, recent studies have tried to improve the efficacy of anti‐angiogenic therapies by blocking EV drug‐resistant molecules. A further study demonstrated that the affinity of EV‐associated VEGF_90K_ has a weakened affinity for bevacizumab, leading to the inability of bevacizumab to effectively inhibit EV‐dependent VEGF receptor activation. However, VEGF_90K_ is released from EVs with the treatment of an Hsp90 inhibitor, thereby restoring sensitivity to bevacizumab.^51^ What's more, the ligand half‐life is significantly increased with the interaction of VEGF_189_ and the surface of EVs, and that associated EV‐VEGF is hard to be neutralized by bevacizumab. The VEGFR2‐neutralizing antibody or VEGFR tyrosine kinase inhibitors can suppress the EV‐VEGF action. Therefore, these inhibitors rather than bevacizumab might be more effective as anti‐tumour therapy for patients who have elevated EV‐VEGF levels.^90^ These findings suggest that the various levels of EV contents should also be considered as an important factor in anti‐angiogenic therapies of selected patients to avoid drug resistance (Figure 3).

## 6 CONCLUSIONS

An increasing number of studies have reported the capacity of EVs to actively regulate the tumour‐associated angiogenic programmes. It is now well established that EVs can carry and transfer proteins and mRNAs/ncRNAs into target cells, which not only help to reveal the angiogenesis and anti‐angiogenesis mechanisms but also created new perspectives on drug targets and drug resistance in anti‐angiogenesis. However, the inclusion of different molecules from different tumour‐derived EVs may be because of bias in the selection of experimental targets.^91^ Studies on EVs are still required to further identify the key EV cargos in angiogenesis and anti‐angiogenesis, and develop additional anti‐angiogenic drugs targeting EVs. More importantly, their potential should be evaluated in clinical trials, especially the combination therapy with current anti‐tumour treatments. Furthermore, for the EVs can be delivered to cells easier with phospholipid bilayer, recent studies are also focusing on using EVs to deliver drugs or synthetic and biological genetic materials to specific target cells,^92^ which also contribute to anti‐angiogenesis therapy.

## CONFLICT OF INTEREST

The authors declared no conflict of interest.

## AUTHOR CONTRIBUTION

**Shuqiong Zhang:** Writing–original draft (equal); Writing–review & editing (equal). **Junyao Yang:** Conceptualization (equal); Funding acquisition (equal); Writing–original draft (equal); Writing‐review & editing (equal). **Lisong Shen:** Conceptualization (lead); Resources (lead); Writing‐original draft (lead); Writing–review & editing (lead).

## Data Availability

Data sharing is not applicable to this article as no new data were created or analysed in this study.
